# The Editorial on the Special Issue “Research on Mycotoxins in Food and Feed: From Detection and Unravelling of Toxicity to Control”

**DOI:** 10.3390/toxins16100435

**Published:** 2024-10-10

**Authors:** Mohamed F. Abdallah, Shupeng Yang, Elisabeth Varga

**Affiliations:** 1 Department of Food Technology, Safety and Health, Ghent University, 9000 Gent, Belgium; 2 Department of Human Biology and Toxicology, Faculty of Medicine, Pharmacy and Biomedical Sciences, University of Mons, 7000 Mons, Belgium; 3 Institute of Food Science and Technology, Chinese Academy of Agricultural Sciences, Beijing 100193, China; yangshupeng@caas.cn; 4 Unit Food Hygiene and Technology, Centre for Food Science and Veterinary Public Health, Clinical Department for Farm Animals and Food System Science, University of Veterinary Medicine, Vienna, 1210 Vienna, Austria; elisabeth.varga@vetmeduni.ac.at; 5 Department of Food Chemistry and Toxicology, Faculty of Chemistry, University of Vienna, 1090 Vienna, Austria

In this Special Issue, several interesting research and review articles were published with the aim of filling in some of the existing knowledge gaps in the field of mycotoxins. In total, ten papers were finally accepted for publication (eight articles and two reviews). The contributions are listed at the end of this Editorial. The accepted research articles aimed to investigate the in vitro toxicity of selected mycotoxins as a mixture with other synthetic food contaminants (Contribution 1 and 2); the efficacy of selected biocontrol agents and different maize hybrids under laboratory and field conditions for the preharvest control of toxigenic fungi (Contribution 3 and 4); the potential use of some natural products in mitigating the toxicity caused by aflatoxins in experimental animals (Contribution 5, 6, and 7); and the simultaneous determination of some emerging mycotoxins in animal feed (Contribution 8). In addition, two scoping review articles covered the global distribution of two toxigenic fungi in maize (Contribution 9) and the reported individual and combined toxicities of aflatoxin B1 (ABF1) and fumonisin B1 (FB1) in vitro (Contribution 10).

As humans are constantly exposed to mixtures of various natural and synthetic food contaminants, it is crucial to investigate the toxic outcomes of binary co-exposure in food [1]. Focusing on a mixture of the emerging *Alternaria* mycotoxin alternariol (AOH) and the process contaminant acrylamide (AA), Crudo et al. applied several well-established assays to investigate the cytotoxic, genotoxic, and mutagenic effects of these two compounds using the hepatic cell line HepG2. Based on their data, there was no immediate cause for concern about synergistic health risks associated with the consumption of foods co-contaminated with AOH and AA (Contribution 2). In another work, the same research group investigated the combinatory toxicological effects of AA and another mycotoxin (deoxynivalenol; DON) by performing experiments based on cytotoxicity, gene transcription, and the expression of major cytochrome P450 (CYP) enzymes in differentiated human hepatic HepaRG cells. The observed cytotoxic effects in the chosen ratios (AA–DON 10:1; 100:1) were driven by DON, with no over-additive effects. The overall conclusion posits that there is no indication of CYP2E1 induction as a critical step in AA bioactivation in the presence of DON (Contribution 1).

An up-to-date overview of the individual and combined toxicities of AFB1 and FB1 was conducted by Chen et al. (Contribution 10). In their work, in vitro studies that investigated the toxicological effects of these two mycotoxins were summarized and discussed. Most studies tested the short-term effect of AFB1 and/or FB1 in HepG2 as a simple hepatic model. A major toxic effect was apoptosis, which is induced by several pathways. As a general conclusion, it could be said that the combined effect probably has a synergistic interaction via the induction of oxidative stress and mitochondria dysfunction through the expression of the Bcl-2 family and p53 proteins (Contribution 10). The relevance of investigating these two toxins results from their common (co-)occurrence in maize and their hepatotoxicity [2].

The best approach suggested to limit human exposure to AFB1 and FB1 is to control the attack and growth of their main producing fungal species such as *Aspergillus flavus* and *Fusarium verticillioides*, respectively [3]. This requires an understanding of their global distribution and their interactions. Therefore, Chen et al., in another work, reviewed the worldwide (co-)occurrence of these two fungal species and how they can interact in maize, which is considered a susceptible crop for *A. flavus* and *F. verticillioides* attack and AFB1 and FB1 production (Contribution 9). Furthermore, an overview of AFB1 and FB1 (co-)occurrence per continent was presented in their review. Interestingly, the interaction of both fungi regarding their growth seemed to be antagonistic under in vitro or in planta conditions. However, the review showed that the (co-)contamination of AFB1 and FB1 has increased in the last decade all over the world. These conclusions were reached from previous studies published between 1980 and 2020. Another suggested strategy to limit the growth of aflatoxigenic fungi in maize during the preharvest stage is the use of maize hybrids with genetic resistance [4]. Barošević et al. evaluated the sensitivity of 20 maize hybrids to *A. flavus* infection and aflatoxin accumulation in field trials (Contribution 3). Through use of a colonized toothpick method for artificial inoculations, they found significant differences in maize hybrids’ susceptibility to *A. flavus* infection and AFB1 production. However, none of the tested hybrids showed complete resistance. Furthermore, the effect of sowing date was seen as a major contributor to the hybrids’ response to the fungal attack. Another ecofriendly solution to inhibit the growth of toxigenic fungi and the production of their associated mycotoxins is the application of effective biocontrol agents such as plant growth-promoting bacteria. This type of beneficial bacteria can play a vital role in managing toxigenic fungi-caused diseases such as *Fusarium* ear rot. A novel endophytic *Pseudescherichia* sp. GSE25 strain was isolated, identified, and tested against *Fusarium graminearum* and the mycotoxin DON in wheat by Gao et al. (Contribution 4). The in vitro testing of this bacterial endophyte showed antifungal activity against *F. graminearum* PH-1 by inhibiting spore germination. Under field conditions, the strain significantly reduced the disease incidence and DON levels by over 60% and 80%, respectively. This strain could be a promising tool as a biofungicide to protect wheat crop from the devastating effect of *F. graminearum* as the strain did not show any hemolytic activity during the biosafety assessments conducted by the authors (Contribution 4).

The impacts of *Chlorella vulgaris* (CLV) and Arabic gum (AG) were assessed in alleviating hepatic aflatoxicosis in quails and male Wistar rats, respectively (Contribution 5 and 6). Elbasuni et al. found that CLV supplementation (1 g/kg diet) ameliorated aflatoxin-induced oxidative stress and inflammatory conditions in 13-week-old Japanese quails (Contribution 5). A similar conclusion was also reported by Ahmed et al. in 15-week-old male Wistar rats after investigating a mixture of aflatoxins with AG (7.5 g/kg b.w) in drinking water (Contribution 6). A random-effects meta-analysis followed by meta-regression was conducted by Weaver et al. and showed that exposure to mycotoxins at or below regulatory guidelines lowered the average feed intake and daily gain in pigs (nursery and grow–finish). This effect was mitigated when supplementation of yeast cell wall extract was added to the feed (Contribution 7).

Finally, the detection and quantification of mycotoxins in different matrices is crucial for ensuring the safety of food and feed [5]. In this regard, a reliable and sensitive ESI +/− LC-MS/MS method was developed and validated for the simultaneous determination of six *Alternaria* mycotoxins (altenuene, alternariol, alternariol monomethyl ether, altertoxin I, tenuazonic acid, and tentoxin) in cattle and sheep feeds (Contribution 8). When the method was applied to analyze feed samples collected from Xinjiang Province in China, four out of the six target *Alternaria* mycotoxins occurred in >40% of the samples, with concentrations ranging between 4 and 551 µg/kg.

Overall, the accepted manuscripts provide new insights into (1) the combined toxicity of mycotoxins as a mixture with other synthetic food contaminants, (2) the biocontrol of toxigenic fungi and the inhibition of mycotoxin production in plants, (3) the potential use of natural products in mitigating mycotoxicosis in animals, and (4) the continuous need for accurate and reliable analytical tools for (emerging) mycotoxin detection in animal feed.
